# Development and Validation of the Observation Checklist Assessing the Hygiene and Sanitation of the Food Preparation Areas in Kota Bharu Kelantan Preschool

**DOI:** 10.7759/cureus.44488

**Published:** 2023-08-31

**Authors:** Mohd Khairul Ashraf Khalid, Nik Rosmawati Nik Husain, Wan Mohd Zahiruddin Wan Mohammad, Norazlin Idris, Natrah Abu Bakar

**Affiliations:** 1 Community Medicine, Universiti Sains Malaysia School of Medical Sciences, Kota Bharu, MYS; 2 Food Safety and Quality Unit, Kelantan State Health Department, Kota Bharu, MYS

**Keywords:** practice, knowledge, checklist, preschools, food preparation area, sanitation, hygiene

## Abstract

Introduction: The current inspection checklist for the assessment of food preparation areas in preschools in Malaysia has not been revised since 2012. The checklist’s content needs to be improved to ensure relevant parameters are covered during preschool inspections.

Objective: The objective of this study was to develop and validate an observation checklist for assessing the hygiene and sanitation of food preparation areas in preschools.

Methodology: The study was conducted in Kota Bharu Kelantan from March 2021 to February 2022. The development of the observation checklist was conducted in four stages: (1) the construction of domains and items from the existing literature, (2) content validation by six experts (using the item-level content validity index (I-CVI) and the scale-level content validity index (S-CVI), (3) face validation by 10 experts (using the item-level face validity index (I-FVI) and the scale-level face validity index (S-FVI)), and (4) reliability analysis (using the intercorrelation coefficient (ICC)). Four assessors performed the reliability analysis at two preschools.

Results: The initial draft of the checklist contained three domains and 57 items: building and facility (10 subdomains and 38 items), process control (four subdomains and 12 items), and food handlers (one subdomain and seven items). The I-CVI scores for building and facility, process control, and food handlers were 0.97, 1.00, and 1.00, respectively, indicating good relevancy of items. The S-CVI value was 1.0 for all domains, showing good relevance of the items. The I-FVI above 0.8 and S-FVI values above 0.9 for all domains imply that the participants easily understood the checklist. The ICC for each domain was 0.847 (95% CI 0.716-0.902) for the building facility and 1.0 for process control and food handler, and the ICC for the three domains combined was 0.848 (95% CI 0.772-0.904). The final validated checklist consists of three domains with 57 items.

Conclusion: The newly developed observation checklist is a valid and reliable tool for assessing the hygiene and sanitation of preschool food preparation areas.

## Introduction

A total of 600 million people worldwide have suffered from foodborne diseases (FBD), causing around 420,000 people to die each year. Of the deaths, 30% occurred among children under five years of age, indicating that children under five years are the most vulnerable population to FBD [1]. Environmental factors account for 24% of the disease burden, and 36% of deaths in children are due to environmental contaminants [2].

According to the Malaysia Ministry of Health (MOH), the food poisoning incidence rate in Malaysia from 2008 to 2018 ranged from 36.17 to 62.47 over the 100,000 population, and the number of cases ranged from 303 to 522 per year [3]. The incidence rate of food poisoning among children ≤ 6 years old ranged from 0.5 to 3.2 per 100,000 from 2015 to 2019, and the number of cases increased from eight to 61 from 2015 to 2018 [4].

Children are more vulnerable to FBD because their immunity levels are less developed as compared to adults. They also lack knowledge of food safety and are unable to control their food selection because food is prepared and served in preschool by the teachers [5]. Furthermore, children spend most of their time in preschools during the day, starting from 7:30 a.m. to 1:00 p.m. (half day) or 5:00 p.m. (full day). In routine preschool management, food is solely prepared and served in the preschools at least three times daily (breakfast, brunch, and lunch) [6].

The hygiene level in the food preparation area is the most critical aspect to be considered because it reflects the safety of the food to be consumed [7]. Foodborne bacteria can grow on most kitchen surfaces, including cutting boards, cloths, sinks, cleaning sponges, and knives [8]. Food particles on kitchen surfaces may serve as reservoirs of bacteria growth if they are not adequately cleaned using soap and water. In addition, food handlers must always practice good hygiene during food preparation to ensure that food is safe to be consumed by their clients, especially the most vulnerable group [9].

Enforcement of the law requires regular monitoring of food preparation areas in preschools to ensure that food safety rules and regulations are followed. Currently, Malaysian preschool inspection is conducted once per year by the law enforcement team or by request of the preschool operators [10]. The checklist used for preschool inspection was developed and validated in 2012 and has not been revised since 2012 [10]. The current checklist only consisted of two domains and six items. The first domain is the kitchen and food preparation area with three items: (i) floor, wall, and ceiling hygiene (slippery/non-slippery floor and clean/dirty); (ii) sink hygiene (clean/dirty and equipped with soap/no soap); and (iii) fridge hygiene (clean/dirty and functioning/not functioning). The second domain is food handler with three items: (i) food source (cooked on-premises/bought from outside premises), (ii) anti-typhoid vaccination status (yes/no), and (iii) food handling course status (yes/no) [10].

Several parameters with a known risk for FBD are not considered in the current checklist, including proper lighting and ventilation of the food preparation area [11]; the food cooling method and storage temperature [12]; food safety practices among food handlers and use of personal protection during food preparation [13,14]; and the availability of food preparation equipment or utensils, separation of the food preparation area, food storage area, garbage management, staff restroom, and the water supply source [14,15]. Therefore, this research aims to develop and validate a checklist to assess the hygiene and sanitation of food preparation areas in preschools.

## Materials and methods

The observation checklist was developed and validated in four stages.

Stage 1: Selection of domains and items

The first stage started with selecting the related domains and items based on previous studies. An extensive literature review was conducted to choose the appropriate domains and items to assess the hygiene and sanitation of food preparation areas in preschools. Three domains were considered: (1) building and facility with 10 subdomains and 38 items, (2) process control with four subdomains and 12 items, and (3) food handlers with one subdomain and seven items. The checklist had three options for the answers, and the scoring methods were not satisfied (0 marks), satisfied (1 mark), and very satisfied (2 marks).

Stage 2: Content validation

The second stage, content validation of the draft checklist, was performed by a panel of six experts: one public health physician, one food quality and safety officer, one communicable disease control officer, one preschool manager, and two preschool teachers. The numbers on the expert panel followed previous studies’ recommendations [16-18].

The expert panel was handed the checklist with information regarding its development, guidelines for the evaluation, and the requirements for performing the analysis. The panel evaluated the constructed items based on the level of relevancy using a four-point scale: 1 - not relevant; 2 - slightly relevant; 3 - moderately relevant; and 4 - very relevant, as recommended by Davis [19]. The panel was asked to analyze and clarify the importance of each domain and item and provide a score for each item. In addition, they could provide suggestions, such as the inclusion or exclusion of each item in the checklist [19,20]. Finally, the content validation results were measured using the item-level content validity index (I-CVI) and the scale-level content validity index (S-CVI).

Stage 3: Face validation

During the third stage, 10 raters performed the face validation of the checklist, as suggested in the literature [21]. The raters evaluated the clarity of the instruction, the language used, and the comprehension of the instruction given in a checklist format [22]. The rating scores were as follows: 1 - clear and understandable and 0 - unclear and confusing. The 10 raters chosen were assistant environmental and health officers: four from the Communicable Disease Control Unit, three from the Environmental Health and Occupational Safety Unit, and three from the Food Quality and Safety Unit. The raters were directly involved in the inspections of preschools and food premises. Finally, the face validation results were measured using the item-level face validity index (I-FVI) and the scale-level face validity index (S-FVI).

Stage 4: Reliability analysis

In the fourth stage, the reliability analysis was performed by calculating each domain’s intercorrelation coefficient (ICC). The reliability analysis used ICC instead of Kappa agreement (Cronbach alpha) due to the outcome of the checklist being quantitatively measured as not satisfactory (0 points), satisfactory (1 point), and very satisfactory (2 points). The ICC is a reliability index that shows the degree of agreement between rater measurements that use a checklist [23]. Four assessors performed the data collection from May 2021 to August 2021 in two preschools located in Kota Bharu Kelantan. They used the same checklist simultaneously, independently, and without conferring during the evaluation. The completed checklists were collected at the end of the assessment, and the final score was then calculated.

The calculated score was entered and analyzed using SPSS version 26.0 software (IBM Corp., Armonk, NY). The reliability analysis was calculated using the reliability test (absolute agreement, two-way mixed-effects model) to calculate the ICC of the three constructed domains. An ICC with a value < 0.5 indicates poor reliability, 0.5-0.75 indicates moderate reliability, 0.75 -0.9 indicates good reliability, and >0.9 indicates excellent reliability [23].

Ethical considerations

The study was approved by the Community Development Department (KEMAS) of Ministry of Rural Development Malaysia (KEMAS.BPAK 620-02/01.01 Jld24(77)), the Department of Planning and Educational Research of Ministry of Education Malaysia (KPM.600-3/2/3-eras (9407)), and the Human Research Ethics Committee of Universiti Sains Malaysia (USM/JEPeM/21020156). Data confidentiality was firmly preserved, and viewing was restricted to the authors and supervisors. The results have been anonymized for reporting and publication.

## Results

Content validation

A panel of six experts conducted the content validation process for the newly developed checklist. The I-CVI scores for each item ranged from 0.83 to 1.0. The S-CVI scores for the building and facility, process control, and food handler domains were 0.99, 1.0, and 1.0, respectively (Tables 1-3). The content validation results indicate good relevancy for assessing those three domains [24].

**Table 1:** Content validity index for domain 1 (building and facility) based on the relevancy rating of items by six experts ^a^E: Expert. ^b^I-CVI: Item-level CVI. ^c^S-CVI/Ave: Scale-level content validity index, averaging method.

Items	E^a^1	E^a^2	E^a^3	E^a^4	E^a^5	E^a^6	Expert in agreement	I-CVI^b^
*1. Structure of the food preparation area*								
a) Clean and suitable floor	1	1	1	1	1	1	6	1.0
b) Clean and suitable wall	1	1	1	1	1	1	6	1.0
c) Clean and suitable ceiling	1	1	1	1	1	1	6	1.0
*2. Food preparation area*								
a) Adequate space for food preparation	1	1	1	1	1	1	6	1.0
b) Adequate lighting	1	1	1	1	1	1	6	1.0
c) Adequate ventilation	1	1	1	1	1	1	6	1.0
*3. Food storage*								
a) Separate storage space for dry and wet food	1	1	1	1	1	1	6	1.0
b) Separate container for cooked food	1	1	1	1	1	1	6	1.0
c) Labeled food container	1	1	1	1	1	1	6	1.0
d) Closed food container	1	1	1	1	1	1	6	1.0
*4. Fridge hygiene*								
a) Clean	1	1	1	1	1	1	6	1.0
b) Food storage temperature following standard	1	1	1	1	1	1	6	1.0
c) Raw and cooked food stored in separate container	1	1	1	1	1	1	6	1.0
d) Cover the food container to avoid contamination from the above container	1	1	1	1	1	1	6	1.0
*5. Sink hygiene*								
a) Clean	1	1	1	1	1	1	6	1.0
b) Adequate water supply	1	1	1	1	1	1	6	1.0
c) Equipped with hand soap	1	1	1	1	1	1	6	1.0
d) Not using rubber hose	1	1	1	1	1	1	6	1.0
e) The sink outlet is equipped with a grease trap	1	1	1	1	1	1	6	1.0
*6. Cooking utensils*								
a) Clean	1	1	1	1	1	1	6	1.0
b) Not broken/corroded	0	1	1	1	1	1	5	0.83
c) Separate container for dry and wet food preparation	1	1	1	1	1	1	6	1.0
d) Separate knife used for raw and cooked food	1	1	1	1	1	1	6	1.0
e) Separate chopping board used for raw and cooked food	1	1	1	1	1	1	6	1.0
*7. Trash handling*								
a) Dustbin placed in a suitable area	1	1	1	1	1	1	6	1.0
b) Clean dustbin	1	1	1	1	1	1	6	1.0
c) Closed dustbin	1	1	1	1	1	1	6	1.0
d) Dustbin equipped with plastic cover	1	1	1	1	1	1	6	1.0
*8. Water source*								
a) Clean, safe, and adequate water supply	1	1	1	1	1	1	6	1.0
9. Toilet hygiene								
a) Clean and no foul-smelling	1	1	1	1	1	1	6	1.0
b) Equipped with adequate water supply	1	1	1	1	1	1	6	1.0
c) Equipped with soap	1	1	1	1	1	1	6	1.0
d) Separated from food preparation, storage, and dining area	1	1	1	1	1	1	6	1.0
*10. Drainage system*								
a) Clean, no foul-smelling	1	1	1	1	1	1	6	1.0
b) Discharge water drained to closed drainage system	1	1	1	1	1	1	6	1.0
c) Discharge water flow not clogged	1	1	1	1	1	1	6	1.0
d) Discharge water disposed to a suitable area	1	1	1	1	1	1	6	1.0
e) Drain equipped with food waste trap	1	1	1	1	1	1	6	1.0
							^c^S-CVI/Ave	0.99

**Table 2:** Content validity index for domain 2 (process control) based on the relevancy rating of items by six experts ^a^E: Expert. ^b^I-CVI: Item-level CVI. ^c^S-CVI/Ave: Scale-level content validity index, averaging method.

Items	E^a^1	E^a^2	E^a^3	E^a^4	E^a^5	E^a^6	Expert in agreement	I-CVI^b^
*1. Organization schedule of the food preparation area*								
a) Cleaning schedule record	1	1	1	1	1	1	6	1.0
b) Food handler training record	1	1	1	1	1	1	6	1.0
c) Anti-typhoid vaccination record	1	1	1	1	1	1	6	1.0
*2. The control of the food preparation process*								
a) Appropriate food thawing method	1	1	1	1	1	1	6	1.0
b) Appropriate temperature for raw food storage	1	1	1	1	1	1	6	1.0
c) Appropriate temperature for cooked food storage	1	1	1	1	1	1	6	1.0
d) Cooked food is served within 4 hours	1	1	1	1	1	1	6	1.0
*3. The hygiene of food preparation*								
a) Raw food is washed properly prior to cooking	1	1	1	1	1	1	6	1.0
b) Raw meat is cleaned within 1 hour	1	1	1	1	1	1	6	1.0
*4. Pest control*								
a) The food preparation area is free from pest	1	1	1	1	1	1	6	1.0
b) Pest control schedule	1	1	1	1	1	1	6	1.0
c) Equipped with pest trap	1	1	1	1	1	1	6	1.0
							^c^S-CVI/Ave	1.0

**Table 3:** Content validity index (S-CVI) for domain 3 (food handler) based on the relevancy rating of items by six experts ^a^E: Expert. ^b^I-CV: Item-level CVI. ^c^S-CVI/Ave: Scale-level content validity index, averaging method.

Items		E^a^1	E^a^2	E^a^3	E^a^4	E^a^5	E^a^6	Expert in agreement	I-CV1^b^
1. Food handler									
a) Underwent medical examination		1	1	1	1	1	1	6	1.0
b) Vaccinated with anti-typhoid vaccine		1	1	1	1	1	1	6	1.0
c) Attended food handler training course		1	1	1	1	1	1	6	1.0
d) Appropriate and clean attire		1	1	1	1	1	1	6	1.0
e) Clean and short hand nails		1	1	1	1	1	1	6	1.0
f) Not wearing jewelry during food handling		1	1	1	1	1	1	6	1.0
g) Wash hands before and after handling food)		1	1	1	1	1	1	6	1.0
								^c^S-CVI/Ave	1.0

Face validation

Ten assistant environmental and health officers conducted the face validation of the hygiene and sanitation observation checklist. The I-FVI value for each item ranged from 0.8 to 1, and the S-FVI values for the building and facility, process control, and food handler domains were 0.99, 0.97, and 1.0, respectively. FVI values above 0.80 are considered acceptable (Tables 4-6) [24,25].

**Table 4:** Face validity index of domain 1 (building and facility) based on relevancy rating of items by 10 PPKP raters ^a^R: Rater. ^b^I-FVI: Item-level FVI. ^c^S-FVI/Ave: Face-level content validity index, averaging method. PPKP: Assistant Environmental Health Officers.

Items		R^a^1	R^a^2	R^a^3	R^a^4	R^a^5	R^a^6	R^a^7	R^a^8	R^a^9	R^a^10	Rater in agreement	I-FVI^b^
*1. Food preparation area structure*													
a) Clean and suitable floor		1	1	1	1	1	1	1	1	1	1	10	1.0
b) Clean and suitable wall		1	1	1	1	1	1	1	1	1	1	10	1.0
c) Clean and suitable ceiling		1	1	1	1	1	1	1	1	1	1	10	1.0
*2. Food preparation area*													
a) Adequate space for food preparation		1	1	1	1	1	1	1	1	1	1	10	1.0
b) Adequate lighting		1	1	1	1	1	1	1	1	1	1	10	1.0
c) Adequate ventilation		1	1	1	1	1	1	1	1	1	1	10	1.0
*3. Food storage area*													
a) Separate storage space for dry and wet food		1	1	1	1	1	1	1	1	1	1	10	1.0
b) Separate container for cooked food		1	1	1	1	1	1	1	1	1	1	10	1.0
c) Labeled food container		1	1	1	1	1	1	1	1	1	1	10	1.0
d) Closed food container		1	1	1	1	1	1	1	1	1	1	10	1.0
*4. Fridge hygiene*													
a) Clean		1	1	1	1	1	1	1	1	1	1	10	1.0
b) Food storage temperature following standard		1	1	1	1	1	1	1	1	1	1	10	1.0
c) Raw and cooked food stored in a separate container		1	1	1	1	1	1	1	1	1	1	10	1.0
d) The food container is covered to prevent contamination from the above container		1	1	1	1	1	1	1	1	1	1	10	1.0
*5. Sink hygiene*													
a) Clean		1	1	1	1	1	1	1	1	1	1	10	1.0
b) Adequate water supply		1	1	1	1	1	1	1	1	1	1	10	1.0
c) Equipped with hand soap		1	1	1	1	1	1	1	1	0	1	9	0.9
d) Not using rubber hose		1	1	1	1	1	1	1	1	1	1	10	1.0
e) The sink outlet is equipped with a grease trap		1	1	1	1	1	1	1	1	1	1	10	1.0
*6. Cooking utensils*													
a) Clean		1	1	1	1	1	1	1	1	1	1	10	1.0
b) Not corroded/unbroken		1	1	1	1	1	1	1	1	1	1	10	1.0
c) Separate container for dry and wet food preparation		1	1	1	1	1	1	1	1	1	1	10	1.0
d) Separate knife used in cutting raw and cooked food		1	1	1	1	1	1	1	1	1	1	10	1.0
e) Separate chopping board used in cutting raw and cooked food		1	1	1	1	1	1	1	1	1	1	10	1.0
*7. Garbage management*													
a) Dustbin placed in a suitable area		1	1	1	1	1	1	1	1	1	1	10	1.0
b) Clean dustbin		1	1	1	1	1	1	1	1	1	1	10	1.0
c) Closed dustbin		1	1	1	1	1	1	1	1	1	1	10	1.0
d) Dustbin equipped with plastic cover		1	1	1	1	1	1	1	1	1	1	10	1.0
*8. Water supply*													
a) Clean, safe, adequate water supply		1	1	1	1	1	1	1	1	1	1	10	1.0
*9. Toilet*													
a) Clean and no foul-smelling		1	1	1	1	1	1	1	1	1	1	10	1.0
b) Equipped with adequate water supply		1	1	0	1	1	1	1	1	1	1	9	0.9
c) Equipped with hand soap		1	1	1	1	1	1	1	1	1	1	10	1.0
d) Separated from food preparation area, storage, and dining area		1	1	1	1	1	1	1	1	1	1	10	1.0
*10. Drainage system*													
a) Clean, no foul-smelling		1	1	1	1	1	1	1	0	1	1	9	0.9
b) Drainage system is closed		1	1	1	1	1	1	1	1	1	1	10	1.0
c) Drain flow not clogged		1	1	1	1	1	1	1	1	1	1	10	1.0
d) Discharge water drain to the appropriate disposal area		1	1	1	1	1	1	1	1	1	1	10	1.0
e) Drain equipped with food waste trap		1	1	1	1	1	1	1	1	1	1	10	1.0
												^c^S-FVI/Ave	0.99

**Table 5:** Face validity index of domain 2 (process control) based on relevancy rating of items by 10 PPKP raters ^a^R: Rater. ^b^I-FVI: Item-level FVI. ^c^S-FVI/Ave: Face-level content validity index, averaging method. PPKP: Assistant Environmental Health Officers.

Items	R^a^1	R^a^2	R^a^3	R^a^4	R^a^5	R^a^6	R^a^7	R^a^8	R^a^9	R^a^10	Rater in agreement	I-FVI^b^
1. Organization schedule												
a) Cleaning schedule record	1	1	1	1	1	1	1	1	1	1	10	1.0
b) Food handler training record	1	1	1	1	1	1	1	1	1	1	10	1.0
c) Anti-typhoid vaccination record	1	1	1	1	1	1	1	1	1	1	10	1.0
2. Process control												
a) Appropriate food thawing method	1	1	1	1	1	1	1	1	1	1	10	1.0
b) Appropriate raw food storage temperature	1	1	1	1	1	1	1	1	1	1	10	1.0
c) Appropriate temperature for cooked food storage	1	1	1	1	1	0	1	1	1	1	9	0.9
d) Cooked food is served within 4 hours	1	1	1	1	1	1	1	1	1	1	10	1.0
3. Process control hygiene												
a) Raw food is properly washed prior to cook	1	1	1	1	1	1	1	1	1	1	10	1.0
b) Raw meat is washed within 1 hour	1	1	1	0	1	0	1	1	1	1	8	0.8
4. Pest control												
a) No pests seen in the food preparation area	1	1	1	1	1	1	1	1	1	1	10	1.0
b) Regular pest control schedule	1	1	1	1	1	1	1	1	1	1	10	1.0
c) Equipped with pest trap	1	1	1	1	1	1	1	1	1	1	10	1.0
											^c^S-FVI/Ave	0.97

**Table 6:** Face validity index (F-CVI) of domain 3 (food handler) based on relevancy rating of items by 10 PPKP raters ^a^R: Rater. ^b^I-FVI: Item-level FVI. ^c^S-FVI/Ave: Face-level content validity index, averaging method. PPKP: Assistant Environmental Health Officers.

Items	R^a^1	R^a^2	R^a^3	R^a^4	R^a^5	R^a^6	R^a^7	R^a^8	R^a^9	R^a^10	Rater in agreement	I-FV1^b^	
*1. Food handler*													
a) Underwent medical checkup	1	1	1	1	1	1	1	1	1	1	10	1.0	
b) Vaccinated with anti-typhoid vaccine	1	1	1	1	1	1	1	1	1	1	10	1.0	
c) Attended food handler training course	1	1	1	1	1	1	1	1	1	1	10	1.0	
d) Appropriate and clean attire	1	1	1	1	1	1	1	1	1	1	10	1.0	
e) Clean and short hand nails	1	1	1	1	1	1	1	1	1	1	10	1.0	
f) Not wearing jewelry during food handling	1	1	1	1	1	1	1	1	1	1	10	1.0	
g) Wash hands before and after handling food	1	1	1	1	1	1	1	1	1	1	10	1.0	
											cS-FVI/Ave	1.0	

The reliability analysis

The intraclass correlation coefficient (ICC) for all three domains combined (building and facility, process handling, and food handlers) was 0.848 (95% CI: 0.772-0.904, p < 0.001). The ICC value indicates good reliability in assessing the building and facility, process control, and food handler domains. According to Koo and Li, the values of ICC between 0.75 and 0.90 show good reliability [24].

Final validated checklist

Since all items are within acceptable values of I-CVI and I-FVI, no item was deleted or added to the checklist. The final validated checklist consisted of three domains and 57 items. The first domain is building and facility (10 subdomains and 38 items), the second domain is process control (four subdomains and 12 items), and the third domain is food handlers (one subdomain and seven items).

## Discussion

Hygiene and sanitation are basic concepts in preventing FBD. To conduct the best assessment of food preparation areas in preschool, an observation checklist needs to cover the holistic aspects of FBD risk. Therefore, the new observation checklist is intended to replace the currently used checklist, which does not include all the relevant parameters.

In the current study, a panel of six experts validated the newly developed observation checklist. The recommended number of experts on a panel to perform content validation should be at least six people and up to 10 people [22-24]. Content validation is valuable to show the relevancy of the construct items in accessing their respective domains. The results of this study showed good item relevancy to assess the three selected domains.

A newly developed checklist or questionnaire is required to assess face validation to determine whether the developed tools are appropriate and feasible for use by the target population. In this study, face validation involved asking the raters to evaluate the simplicity of the instructions, the language used in the assessment list, and their understanding of the instructions, as suggested by Yusoff [22]. The S-FVI averages for the three domains of building and facility, process control, and food handler ranged from 0.97 to 1.0, indicating acceptable values [21,24-27].

The ICC is a reliability index used to measure the degree of correlation and agreement between inter-rater measurements of the developed checklist. According to Koo and Li [24], the minimum ICC should be ≥0.5. If the ICC value is <0.5, this indicates poor reliability [23]. However, Bravo and Potvin suggest a higher ICC value of ≥0.8 to demonstrate the good reliability of the assessed tool [26]. The combined ICC for the three construct domains showed excellent reliability.

The final observation checklist to assess the hygiene and sanitation of food preparation areas in preschool consists of three domains and 57 construct items. The construct items are valid for measuring three essential domains. Therefore, the newly developed checklist is a valid and reliable tool for assessing the hygiene and sanitation of preschool food preparation areas that cover more parameters than the current checklist (TASKA-4/KPAS/KKM/1/2012) used for preschool inspections by the MOH health inspectors. The parameters added in the newly developed checklist under the building and facility domain are the subdomains of food storage, cooking utensils, garbage management, water sources, toilet hygiene, and the drainage system. The other domain added in the new checklist is control of the food preparation process (i.e., food handling, food storage, and food serving), which was not available in the previous checklist. These parameters should be monitored in food preparation areas to prevent the development of FBD.

Health officers who routinely conduct preschool inspections and assessments to ensure the safe preparation of food can use this newly developed checklist as an inspection tool. This checklist can provide and generate a scoring level of hygiene and sanitation for the assessed food preparation area in preschools. This information is essential for monitoring and evaluating preschool owners’ corrective improvements taken following recommendations given by the health officer. The assessors will be given additional education and training in using the new tool in order to familiarize the usage and to get a better understanding of the parameters covered in the newly developed checklist. Once they are familiarized with the new tools, the inspection will become much easier to be done.

COVID-19 and movement control order 3.0 were the main challenges in conducting this study, which limited the recruitment of many preschools for data collection. In addition, due to the limited study duration, the sample size was smaller than estimated.

## Conclusions

This newly developed checklist is valid and reliable for evaluating the hygiene and sanitation of preschool food preparation areas. The checklist has been tested using content validation by a panel of six experts, face validation by 10 experienced raters, and reliability analysis by examining the ICC. The final checklist comprises 57 items under three domains, and the wording of the checklist items is appropriately comprehended with clear instructions.
